# Scapho-luno-capitate fusion with proximal lunate articular surface preservation for management of grade IIIA Kienböck’s disease: a prospective case series

**DOI:** 10.1186/s10195-023-00703-9

**Published:** 2023-05-18

**Authors:** Ahmed Shams, Mohamed Ahmed Samy, Mohamed Kamal Mesregah, Ahmed Abdelazim Abosalem

**Affiliations:** Department of Orthopaedic Surgery, Faculty of Medicine, Menoufia University, Shebin-El-Kom, Menoufia Egypt

**Keywords:** Kienböck’s disease, Lunate avascular necrosis, Limited carpal fusion, Iliac crest graft, Scapho-luno-capitate fusion, K-wires

## Abstract

**Background:**

Kienböck’s disease is idiopathic lunate avascular necrosis, which may lead to lunate collapse, abnormal carpal motion and wrist arthritis. The current study aimed to assess the outcomes of treating stage IIIA Kienböck’s disease by a novel technique of limited carpal fusion via partial lunate excision with preservation of the proximal lunate surface and scapho-luno-capitate (SLC) fusion.

**Materials and methods:**

We conducted a prospective study of patients with grade IIIA Kienböck’s disease managed with a novel technique of limited carpal fusion comprising SLC fusion with preservation of the proximal lunate articular cartilage. Autologous iliac crest bone grafting and K-wires fixation were used to enhance the osteosynthesis of the SLC fusion. The minimum follow-up period was 1 year. A visual analog scale (VAS) and the Mayo Wrist Score were utilized for the evaluation of patient residual pain and functional assessment, respectively. A digital Smedley dynamometer was used to measure the grip strength. The modified carpal height ratio (MCHR) was used for monitoring carpal collapse. The radioscaphoid angle, scapholunate angle, and the modified carpal-ulnar distance ratio were used for the assessment of carpal bones alignment and ulnar translocation of carpal bones.

**Results:**

This study included 20 patients with a mean age of 27.9 ± 5.5 years. At the last follow-up, the mean range of flexion/extension range of motion (% of normal side) improved from 52.8 ± 5.4% to 65.7 ± 11.1%, *P* = 0.002, the mean grip strength (% of normal side) improved from 54.6 ± 11.8% to 88.3 ± 12.4%, *P* = 0.001, the mean Mayo Wrist Score improved from 41.5 ± 8.2 to 81 ± 9.2, *P* = 0.002, and the mean VAS score reduced from 6.1 ± 1.6 to 0.6 ± 0.4, *P* = 0.004. The mean follow-up MCHR improved from 1.46 ± 0.11 to 1.59 ± 0.34, *P* = 0.112. The mean radioscaphoid angle improved from 63 ± 10º to 49 ± 6º, *P* = 0.011. The mean scapholunate angle increased from 32 ± 6º to 47 ± 8º, *P* = 0.004. The mean modified carpal-ulnar distance ratio was preserved and none of the patients developed ulnar translocation of the carpal bones. Radiological union was achieved in all patients.

**Conclusions:**

Scapho-luno-capitate fusion with partial lunate excision and preservation of the proximal lunate surface is a valuable option for treating stage IIIA Kienböck’s disease, with satisfactory outcomes.

*Level of evidence* Level IV.

*Trial registration* Not applicable.

## Introduction

Kienböck’s disease is characterized by lunate avascular necrosis with rare spontaneous healing, which may lead to lunate collapse, abnormal carpal motion, and degenerative wrist arthritis [1, 2]. There is still no clear cause for Kienböck’s disease, but a number of intrinsic and extrinsic factors have been proposed [1, 3]. Several studies suggested that lunate osteonecrosis occurs due to increased intraosseous pressure from intraosseous venous thrombosis as a result of anatomical, biological, inflammatory, immune, or coagulation disorders [4, 5]. Males between the ages of 20 and 40 are most commonly affected by this disease [6].

Kienböck’s disease is clinically suspected when there is dorsal wrist pain over the lunate that is sometimes combined with reduced range of motion and weak grip strength [7]. Plain X-rays and magnetic resonance imaging (MRI) are required for diagnosis [8, 9].

The Lichtman classification [10] is widely used to classify this disease, with stage IIIA being lunate fragmentation and collapse with decreased carpal height.

Kienböck’s disease is treated according to its stage at the time of presentation, with no gold standard treatment option [11, 12]. Stage IIIA can be treated with lunate excision to remove the source of pain and limited carpal fusion, including scapho-trapezio-trapezoid (STT), scapho-capitate (SC), and capito-hamate (CH) arthrodesis to modulate the load transmission through the carpus bone [13–16].

The procedure of lunate excision and SC fusion was first reported by Pisano et al. [17], with a proposed biomechanical hypothesis of a reduction of axial loading through the radio-lunate and luno-capitate joints and an increase of axial loading through the radio-scaphoid joint; which may lead to osteoarthritis [18].

In the current study, we sought to assess the functional and radiological outcomes of the management of stage IIIA Kienböck’s disease by a novel technique of limited carpal fusion through partial lunate excision with preservation of the proximal surface of lunate and scapho-luno-capitate (SLC) fusion. Our proposed hypothesis is that this technique can offer more balanced axial load transmission through the radio-lunate and radio-scaphoid joints.

## Materials and methods

### Patient selection

This prospective study included 20 patients with grade IIIA Kienböck’s disease treated with a novel technique of limited carpal fusion comprising SLC fusion with preservation of the proximal lunate articular cartilage, from March 2018 to March 2021 in a University Hospital. Written consent was taken preoperatively from all patients. Our institutional review boards (IRB) and ethics committee approved the study. The minimum follow-up period was 1 year.

The inclusion criteria were patients from 20 to 60 years old with Lichtman grade IIIA Kienböck’s disease. Patients with other Kienböck’s disease grades, damaged proximal lunate articular surface, neurovascular disorders, or failed previous surgery were excluded.

### Preoperative evaluation

A detailed history was obtained from each patient, including onset, course, duration of pain, occupation, difficulty working, dominant hand, history of trauma, and any previous management.

A local examination was performed to assess the tenderness, swelling, and range of wrist movements. The preoperative flexion/extension range of motion was measured. A digital Smedley dynamometer was used to measure the grip strength. The average of three grips for both hands was recorded, with 15-s intervals between each grip. Each grip lasted for at least 3 s. The mean grip strength of the affected hand was recorded as a percentage of the normal hand.

Preoperative anteroposterior and lateral plain wrist X-rays were obtained to determine ulnar variance and the stage of the disease. The modified carpal height ratio, radioscaphoid angle, and scapholunate angle were also measured. The modified carpal-ulnar distance ratio was used to assess the ulnar translocation of the carpal bones. CT and MRI were done for accurate staging of the disease and proper evaluation of the articular cartilage of the lunate. Routine preoperative laboratory investigations were done for all patients.

### Surgical technique

Patients were placed supine with the affected wrist over a radiolucent arm board. General anesthesia was used in all patients. The iliac crest was exposed and draped to harvest the iliac crest bone graft.

A pneumatic tourniquet was applied over smooth padding at mid-arm. After sterilization and draping of the affected limb, a sterile Esmarch bandage was used to exsanguinate the limb, followed by inflation of the pneumatic tourniquet to a pressure of 100 mmHg more than the systolic blood pressure.

A dorsal and slightly radial longitudinal incision was made midway between Lister’s tubercle and the radial styloid process, extending 4–5 cm distally. The subcutaneous tissue was dissected down to the extensor retinaculum, creating radial and ulnar skin flaps. The extensor retinaculum of the 3rd extensor compartment was incised longitudinally with retraction of the extensor pollicis longus radially. The contents of the 4th compartment were subperiosteally elevated and retracted ulnarly. The posterior interosseous nerve in the 4th compartment floor was dissected and excision of 1.5 cm of its length was done with cauterization, and crushing of its proximal end for posterior wrist denervation. The wrist capsule was opened in a ligament-splitting fashion with a radially based flap through bisection of the dorsal intercarpal and dorsal radiocarpal ligaments. The capsulotomy was also extended by incision along the dorsal rim of the distal radius to the radial styloid process. Then, the articular surface of the scaphoid and capitate at the SC joint was decorticated down to the cancellous bone. The distal avascular portion of the lunate was removed with preservation of the healthy proximal articular cartilage and its underlying cancellous bone. Under C-arm guidance, axial traction was applied to the middle finger until correction of the scaphoid rotation, to restore the normal carpal height, and the modified carpal height was measured.

**Fig. 1:**
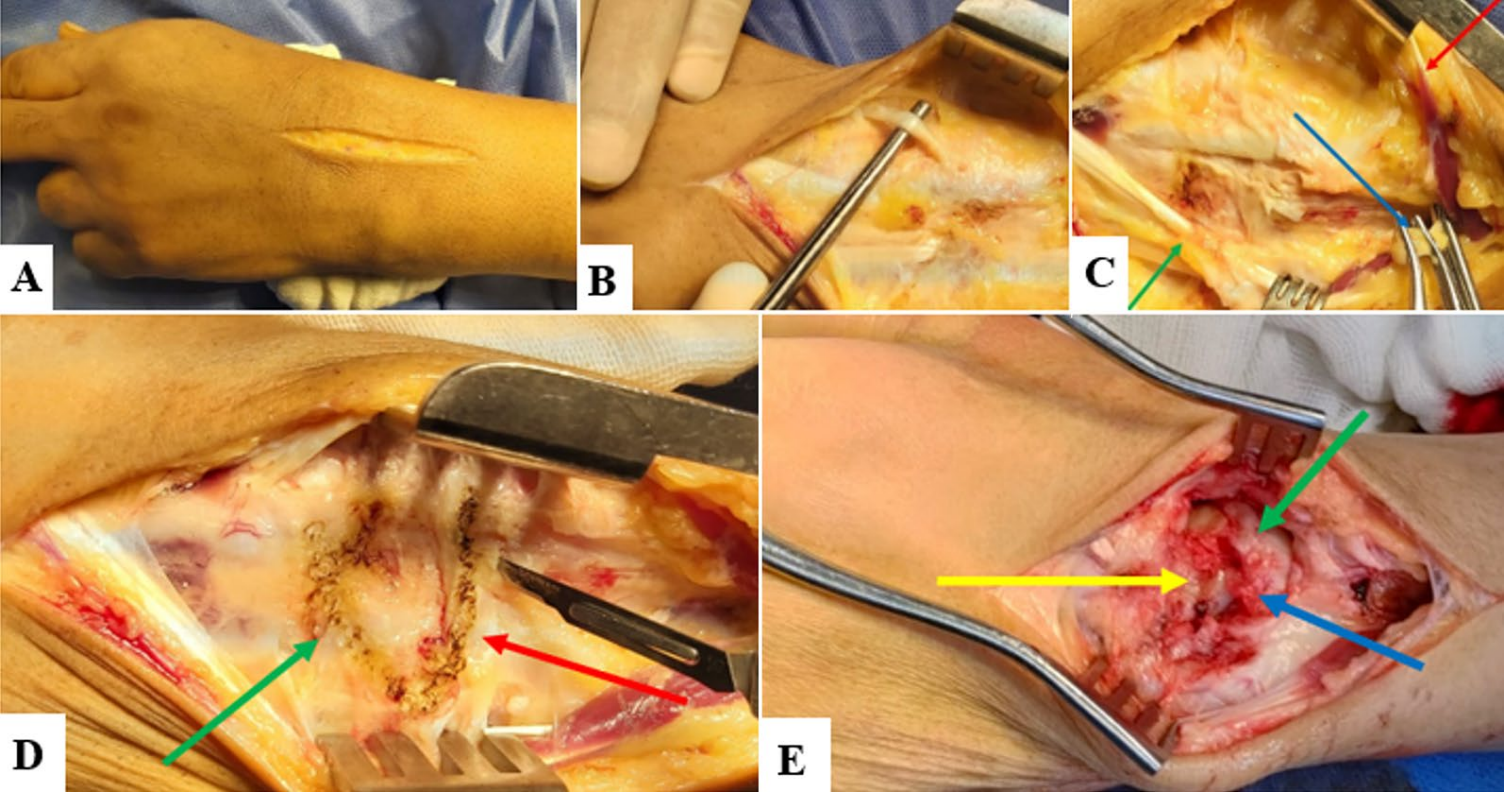
**A** Skin incision. **B** Identification of extensor pollicis longus for incision of the extensor retinaculum of the third extensor compartment. **C** The contents of the fourth compartment were subperiosteally elevated and retracted ulnarly (*green arrow*), while the extensor pollicis longus was retracted radially (*red arrow*). The posterior interosseous nerve (*blue arrow*) was dissected, 1.5 cm of its length was excised and cauterized, and its proximal end was crushed for posterior wrist denervation. **D** The wrist capsule was opened in a ligament-splitting fashion with a radially based flap through bisection of the dorsal intercarpal (*green arrow*) and dorsal radiocarpal ligaments (*red arrow*). **E** Bone grafting of the decorticated articular surface of the scaphoid (*green arrow*), capitate (*yellow arrow*) and the remaining portion of the proximal lunate (*blue arrow*)

An autologous iliac crest bone graft was then applied between scaphoid, lunate, and capitate bones, Fig. 1. Following this, two 1.4-mm K-wires were utilized for fixation of the scapho-capitate junction, restoring their anatomical relation and controlling rotation, and another 1.4-mm K-wire was utilized to fix the lunate to the capitate bones. The K-wires were bent over, cut, and buried under the skin. Deflation of the pneumatic tourniquet followed by proper hemostasis was done. Careful repair of the wrist capsule and extensor retinaculum using 0/2 Vicryl sutures was done. The skin was closed using subcuticular absorbable sutures.

### Postoperative care and functional assessment

**Fig. 2:**
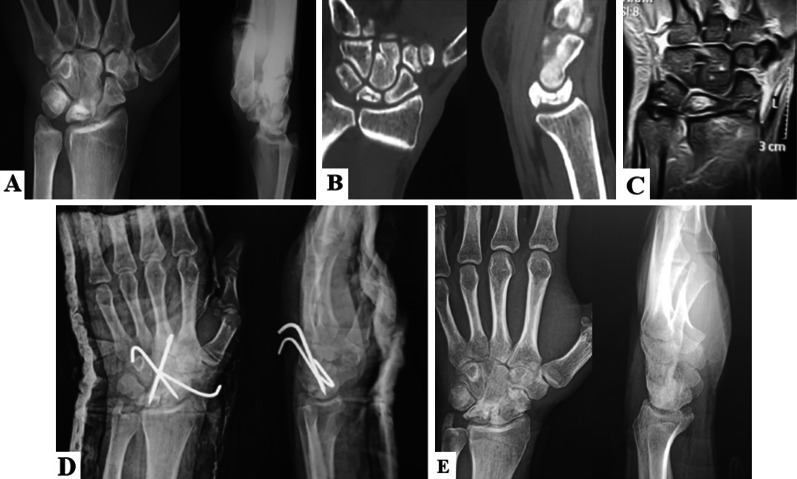
A case of a male patient, 33 years old, with grade IIIA Kienböck’s disease treated with partial lunate excision and scapho-luno-capitate fusion. **A** Preoperative X-rays, anteroposterior and lateral views, showing avascular necrosis and collapse of the lunate. **B** Preoperative coronal and sagittal CT scans showing collapse of the lunate. **C** Preoperative MRI showing intact proximal articular cartilage of the lunate. **D** Immediate postoperative X-rays, anteroposterior and lateral views. **E** One-year follow-up X-rays, anteroposterior and lateral views, showing complete scapho-luno-capitate fusion

A below-elbow splint was applied postoperatively with the encouragement of early mobilization of the fingers. Sutures were removed 2 weeks postoperatively with the application of a short arm cast. K-wires were removed in the outpatient clinic using local anesthesia after achieving complete radiological union. Active range of motion and physiotherapy were commenced. Patients were followed up clinically and radiographically at 2 weeks, 8 weeks, 3 months, and 1 year postoperatively, Fig. 2.

The Mayo Wrist Score [19, 20] (0–100 points score), was used for functional assessment. The visual analog scale (VAS) [21] was used to measure pain before and after the surgical intervention. The modified carpal height ratio (MCHR) [22] was used to monitor carpal collapse. The radioscaphoid angle, scapholunate angle, and the modified carpal-ulnar distance ratio were used for the assessment of carpal bones alignment and ulnar translocation of carpal bones.

### Statistical analysis

Data were tabulated and analyzed using IBM SPSS (Statistical Package for Social Science) version 23. Qualitative data were described as number (*n*) and percent (%), and quantitative data were expressed as mean ± SD and range. The comparison of qualitative data was made using the chi-square test. The comparison of quantitative data was made using the Wilcoxon signed-rank test. The significance level was set at a *P*-value of less than 0.05.

## Results

### Demographics and baseline characteristics

This study included 20 patients, with a mean age of 27.9 ± 5.5 (range, 21–37) years. The demographic data are presented in Table 1.

**Table 1:** Demographics and baseline characteristics of the included patients

Characteristics	Value (*n* = 20)
Age, years (mean ± SD)	27.9 ± 5.5
Gender (*n*, %)	
Male	11 (55%)
Female	9 (45%)
Affected hand (n, %)	
Dominant	13 (65%)
Non dominant	7 (35%)
Ulnar variance (*n*, %)	
Neutral	8 (40%)
Negative	12 (60%)
Occupation (*n*, %)	
Manual worker	9 (45%)
Housewife	7 (35%)
Teacher	2 (10%)
Driver	1 (5%)
Student	1 (5%)

The mean negative ulnar variance was − 2.9 ± 1.6 mm. The mean preoperative flexion/extension range of motion (% of normal side) was 52.8 ± 5.4 (range, 45–60) %. The mean preoperative grip strength (% of contralateral side normal function) was 54.6 ± 11.8 (range, 35–70) %.

The mean preoperative Mayo Wrist Score was 41.5 ± 8.2 (range, 30–55). The mean preoperative VAS score was 6.1 ± 1.6 (range, 4–9). Radiologically, the average preoperative MCHR was 1.46 ± 0.11, which was less than the normal range of 1.51 to 1.61. The mean preoperative radioscaphoid angle was 63 ± 10º. The mean preoperative scapholunate angle was 32 ± 6º. The mean operative time was 88 ± 10.1 (range, 75–105) min. The mean preoperative modified carpal-ulnar distance ratio was 0.67 ± 0.14.

### Functional and radiological outcomes

The mean follow-up period was 18.1 ± 4.7 (range, 12–26) months. At the last follow-up, the mean flexion/extension range of motion (% of normal side) improved to 65.7 ± 11.1 (range, 50–79) %, *P* = 0.002, the mean grip strength (% of normal side) improved to 88.3 ± 12.4 (range, 75–100) %, *P* = 0.001, the mean Mayo Wrist Score improved to 81 ± 9.2 (range, 65–95), *P* = 0.002, and the mean VAS score decreased to 0.6 ± 0.4 (range, 0–1), *P* = 0.004.

Radiologically, the mean MCHR improved to 1.59 ± 0.34 (range, 1.56–1.64), *P* = 0.112. The mean radioscaphoid angle improved significantly to 49 ± 6º, *P* = 0.011. The mean scapholunate angle improved significantly to 47 ± 8º, *P* = 0.004. The mean last-follow-up modified carpal-ulnar distance ratio was comparable to the preoperative ratio, *P* = 0.86, Table 2.

**Table 2:** Preoperative and postoperative clinical and radiological outcome measures

Characteristics	Preoperative	Last follow-up	*P*-value
Flexion/extension ROM, % of normal side, mean ± SD	52.8 ± 5.4%	65.7 ± 11.1%	0.002
Grip strength, % of normal side, mean ± SD	54.6 ± 11.8%	88.3 ± 12.4%	0.001
Mayo Wrist Score, mean ± SD	41.5 ± 8.2	81 ± 9.2	0.002
VAS score, mean ± SD	6.1 ± 1.6	0.6 ± 0.4	0.004
MCHR, mean ± SD	1.46 ± 0.11	1.59 ± 0.34	0.112
Radioscaphoid angle, mean ± SD	63 ± 10º	49 ± 6º	0.011
Scapholunate angle, mean ± SD	32 ± 6º	47 ± 8º	0.004
Modified carpal-ulnar distance ratio	0.67 ± 0.14	0.68 ± 0.15	0.86

*ROM* range of motion; *VAS* visual analog scale; *MCHR* modified carpal height ratio

One patient had persistent pain that was not improved by analgesics, and another surgical procedure was done in the form of total wrist arthrodesis to alleviate the pain. The mean union time was 8.7 ± 4.2 (range, 5–20) weeks. Only one patient had delayed union until 20 weeks and required a longer period of immobilization until achieving union. By the end of the follow-up period, all patients had achieved union and none developed radiocarpal osteoarthritis or ulnar translocation of the carpus.

## Discussion

Kienböck’s disease is a rare disease that was first described about a century ago [23]. The optimal treatment option for Kienböck’s disease with lunate collapse without osteoarthritis (Lichtman stage III) is debatable [14]. Lunate collapse and fragmentation are believed to occur as a consequence of excessive forces across the lunate. Limited carpal fusion aims to unload the lunate, provide an adequate painless wrist function, and delay the development of radio-carpal and middle carpal arthritis [24, 25]. Lunate excision and SC or STT arthrodesis are the most used form of limited carpal fusion for treating the disease [13, 18].

Biomechanical studies have shown that SC fusion reduces the load across the radio-lunate and luno-capitate joints while increasing the load across the radio-scaphoid joint [26, 27].

In this study, we evaluated the outcomes of treating stage IIIA Kienböck’s disease by a novel technique of limited carpal fusion, including SLC fusion. We did partial lunate excision with preservation of the proximal lunate surface to preserve the radio-lunate joint, which could balance axial load transmission through radio-lunate and radio-scaphoid joints. At the last follow-up, this technique resulted in significant improvement in range of motion, grip strength, and Mayo Wrist Score, with a significant reduction in VAS score. Also, no carpal collapse was observed. Only one patient had delayed union until 20 weeks, but at the end of the follow-up period, all patients achieved union.

With the same principle of balancing axial load transmission through radio-lunate and radio-scaphoid joints, the Graner II procedure included complete lunate excision and its replacement with the head of the capitate through capitate lengthening so that the articular surface of the head of the capitate articulated with the lunate surface of the radius. To prevent intercarpal instability following the lunate excision, intercarpal fusion between all carpal bones except the trapezium and pisiform was done [28, 29]. However, this procedure had several reported complications, including osteonecrosis and nonunion of the head of the capitate, and long-term arthritis [29–32].

In our study, at the final follow-up period, the mean hand grip strength was 88.3 ± 12.4% of the contralateral side, which was better than that of Collon et al. [14] with 76% and Charee et al. [18] with 74% strength of the contralateral side. These studies evaluated the outcomes of lunate excision and SC fusion for the management of Lichtman stage III Kienböck’s disease [14, 18].

In our study, all patients had complete SLC fusion at the last follow-up, while the rate of nonunion was 23% in Collon et al. [14], 6% in Charee et al. [18], and 10% in Luegmair et al. [33]. The achievement of full union in our study was attributed to abundant cancellous iliac crest bone grafting and the increased fusion mass between the three bones.

The disease is more likely to develop in patients with negative ulnar variance [24]. In our study, 60% of patients had negative ulnar variance.

Joint leveling surgeries could relieve the symptoms when ulnar variance is negative [34]. However, in stage IIIA, the lunate begins to collapse, and the condition would progress to proximal migration of the capitate and scaphoid rotational instability in stage IIIB. Therefore, limited wrist arthrodesis is recommended once the patient presents with stage III [14, 34].

Limited carpal fusion prevents scaphoid rotatory instability and maintains wrist stability [2]. Various types of limited carpal fusion, including SC, STT, and CH have been described [11]. SC and STT arthrodesis shifts loads from the radio-lunate joint to the radio-scaphoid joint, in addition to reducing force transmission at the luno-capitate joint [35].

During normal power grip, the scaphoid receives approximately 50% of the total load, followed by the lunate at 35%, and the triquetrum at 15% [36]. Lunate loads are then transmitted partly to the radius and partly to the triangular fibrocartilage complex (TFCC) [36]. In our study, we aimed to preserve the proximal surface of the lunate which, theoretically, can maintain balanced axial load transmission through the radio-lunate and radio-scaphoid joints instead of the whole load being transmitted through the radio-scaphoid joint.

Limited carpal fusion is traditionally accompanied by lunate excision, and a few reports in the literature describe SC fusion without lunate excision [14, 37]. It is still questionable whether lunate excision improves wrist function, and whether it is mandatory [14]. Sennwald et al. [37] reported that SC arthrodesis without lunate excision in 11 patients resulted in complete pain relief in 10 patients. Rhee et al. [38] stated that lunate excision might predispose to evolving carpal collapse and ulnar translocation of carpal bones. At present, there is no evidence to support excising the lunate during limited carpal fusion, and it makes the procedure more challenging without improving the results [14].

This study has some limitations, including the relatively small number of patients, the relatively short follow-up period, and the absence of a comparative control group. Future randomized clinical trials comparing our technique with other types of limited carpal fusion, with long-term follow-up, are needed to properly evaluate the functional and radiological outcomes. Moreover, a biomechanical study is needed to estimate the load transmission through the radio-lunate and radio-scaphoid joints following SLC fusion.

## Conclusion

Scapho-luno-capitate fusion with partial lunate excision and preservation of the proximal lunate surface is a reliable technique for the management of stage IIIA Kienböck’s disease, with satisfactory functional and radiological outcomes, high union rates, and negligible complications.

## Data Availability

The dataset analyzed in this study is available from the corresponding author on request.
